# Symptom burden, psychosocial distress and palliative care needs in heart failure – A cross-sectional explorative pilot study

**DOI:** 10.1007/s00392-022-02017-y

**Published:** 2022-04-14

**Authors:** F. Strangl, E. Ischanow, A. Ullrich, K. Oechsle, N. Fluschnik, C. Magnussen, D. Knappe, H. Grahn, S. Blankenberg, C. Bokemeyer, P. Kirchhof, M. Rybczynski

**Affiliations:** 1 Department for Cardiology, University Heart and Vascular Center Hamburg, Martinistr. 52, 20246 Hamburg, Germany; 2 Department for Oncology, University Cancer Center Hamburg, Hamburg, Germany; 3 German Center for Cardiovascular Research (DZHK), Partner Site, Hamburg/ Kiel/ Luebeck, Germany

**Keywords:** Heart failure, Palliative care, Symptom burden, Psychosocial distress

## Abstract

**Background:**

Beyond guideline-directed treatments aimed at improving cardiac function and prognosis in heart failure (HF), patient-reported outcomes have gained attention.

**Purpose:**

Using a cross-sectional approach, we assessed symptom burden, psychosocial distress, and potential palliative care (PC) needs in patients with advanced stages of HF.

**Methods:**

At a large tertiary care center, we enrolled HF patients in an exploratory pilot study. Symptom burden and psychosocial distress were assessed using the MIDOS (Minimal Documentation System for Patients in PC) questionnaire and the Distress Thermometer (DT), respectively. The 4-item Patient Health Questionnaire (PHQ-4) was used to screen for anxiety and depression. To assess PC needs, physicians used the “Palliative Care Screening Tool for HF Patients”.

**Results:**

We included 259 patients, of whom 137 (53%) were enrolled at the Heart Failure Unit (HFU), and 122 (47%) at the outpatient clinic (OC). Mean age was 63 years, 72% were male. New York Heart Association class III or IV symptoms were present in 56%. With a mean 5-year survival 64% (HFU) vs. 69% (OC) calculated by the Seattle Heart Failure Model, estimated prognosis was comparatively good. Symptom burden (MIDOS score 8.0 vs. 5.4, max. 30 points, *p* < 0.001) and level of distress (DT score 6.0 vs. 4.8, max. 10 points, *p* < 0.001) were higher in hospitalised patients. Clinically relevant distress was detected in the majority of patients (HFU 76% vs. OC 57%, *p* = 0.001), and more than one third exhibited at least mild symptoms of depression or anxiety. Screening for PC needs revealed 82% of in- and 52% of outpatients fulfil criteria for specialized palliative support.

**Conclusion:**

Despite a good prognosis, we found multiple undetected and unaddressed needs in an advanced HF cohort. This study’s tools and screening results may help to early explore these needs, to further improve integrated HF care.

**Graphical abstract:**

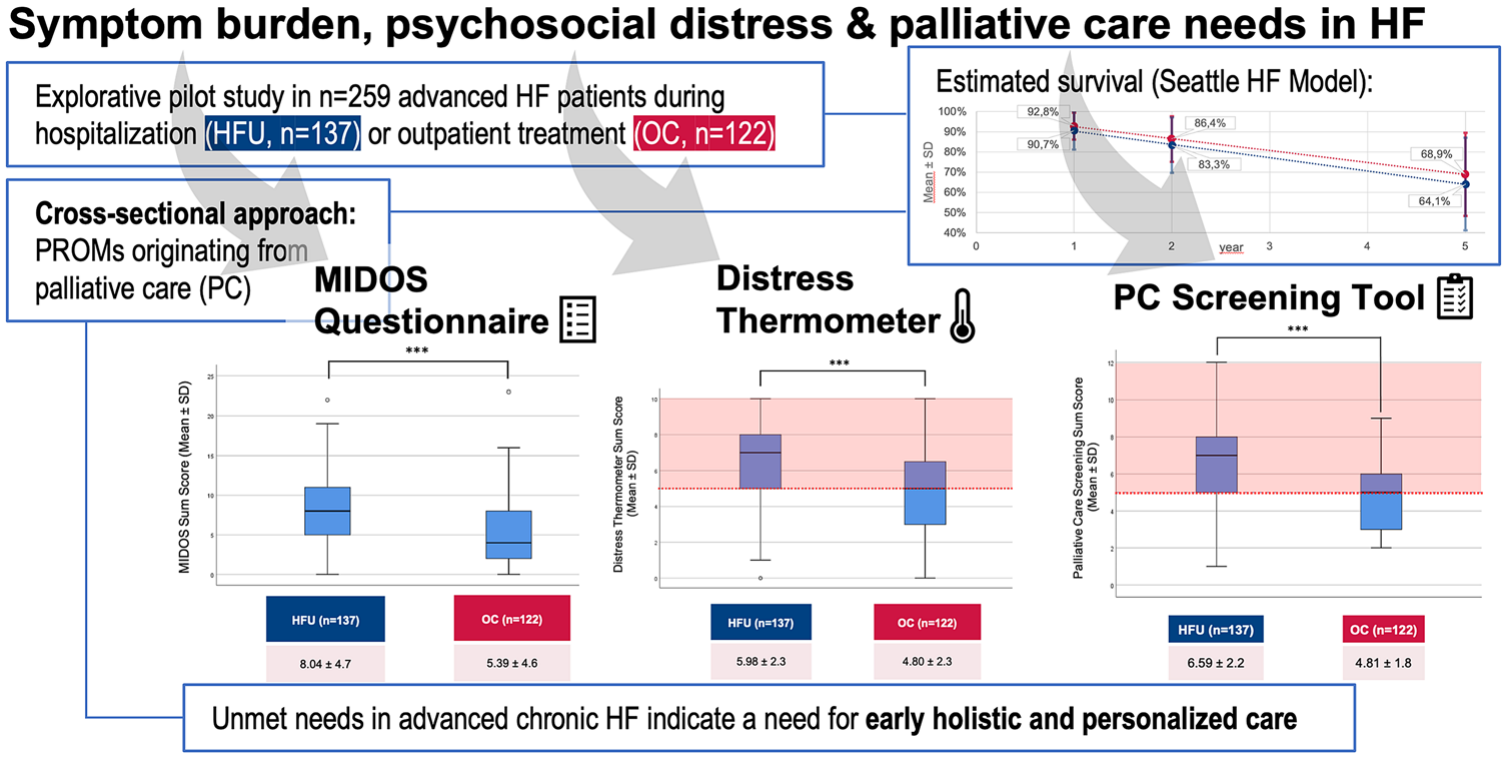

## Introduction

The worldwide disease burden of heart failure (HF), a “global pandemic” [1, 2], is enormous [3], and its prevalence, due to the growth and ageing of the population, is expected to further increase in the coming decades [4, 5]. Especially in elderly patients and advanced stages of HF, morbidity and disability are high [6, 7], as is the caregiver burden [8]. Comorbidities may further compromise quality of life (QoL), as every second HF patient suffers from more than 5 major comorbidities [9].

As the health-related QoL is of paramount importance for patients with advanced HF, many of whom would trade a longer life span for better quality [10, 11], and also affects prognosis [7], patient-reported outcomes (PRO) have gained attention as valuable instruments in guiding HF management [12]. HF specialists have been calling for a comprehensive, if required interdisciplinary care approach tailored to the individual’s needs [13].

These treatments and aid beyond evidence-based HF therapies have been referred to as “palliative care” (PC), as this approach is aimed at improving QoL of patients and their families facing “problems of physical, psychosocial or spiritual nature” [13, 14]. Instead of being tantamount to end-of-life care, a popular misconception, all patients with serious illnesses may benefit from PC also earlier than in the last year of life [15, 16]. The need for PC for chronic diseases may even be greater than for patients suffering from malignancies [15]. Many major cardiovascular society guidelines give recommendations for interdisciplinary PC in advanced HF [17–20]. Primary PC should be applied by treating HF specialists or cardiologists and includes, e.g., pain control, psychosocial care, advanced care planning, identification of goals of care or support for complex treatment and decision-making [21]. PC specialists should be involved in particularly severe or complex situations [21].

The evidence for PC in HF is still in its infancy and is largely drawn from observational studies carrying a risk for subjective and objective bias [21–23], although benefits of PC interventions in this context have recently been demonstrated in randomized controlled trials [24, 25]. As more data on symptom burden, psychosocial distress, and potential PC needs in advanced HF are required to improve this aspect of integrated HF care, we conducted this explorative pilot study using a cross-sectional approach.

## Methods

### Study cohort

The study population consisted of HF patients referred for further treatment evaluation (including implantation of left ventricular assist devices (LVAD) and heart transplantation) to our HF outpatient clinic (OC), and HF patients hospitalised for a worsening HF event at the HF unit (HFU), a specialised intermediate care ward at the University Heart and Vascular Center Hamburg-Eppendorf, a large tertiary care centre in Hamburg, Germany.

Enrollment started in September 2016 and ended in September 2019. All patients aged ≥ 18 years with sufficient cognitive function and adequate knowledge of the German language were considered eligible to participate. Patients on LVAD support and heart transplant recipients were not included. Written informed consent was obtained from all participants. HF treatment was optimized according to current guideline recommendations. The local ethics committee of the General Medical Council of Hamburg, Germany, approved the study protocol (PV 5010).

### Clinical assessment

Clinical variables including age, sex, type of cardiomyopathy, non-cardiac comorbidities (namely arterial hypertension, diabetes, obesity, chronic renal failure, peripheral artery disease, malignancies, pulmonary diseases, neurologic comorbidities, hypothyroidism, hyperthyroidism, psychiatric/psychological comorbidities, nicotine abuse, and alcohol abuse) and cardiac history, as well as clinical findings on presentation such as the New York Heart Association (NYHA) class, were assessed. For a better understanding of HF severity and the stage in the clinical course, we used the Seattle Heart Failure Model [26] (SHFM), a multivariate risk model including clinical status, pharmacological therapy, and device characteristics as well as laboratory parameters, to estimate mean 1-, 2- and 5-year survival in each group.

### Patient-reported outcome measures

Assessments were performed by trained study personnel. Owing to the explorative approach, there were no pre-specified timepoints for study assessments.

The MIDOS survey [27] (Minimal Documentation System For Palliative Care) served as a self-assessment tool for the symptom burden. The questionnaire, originating from PC, measures the intensity of ten frequent symptoms: Pain, nausea, emesis, dyspnea, constipation, weakness, loss of appetite, fatigue, sadness, and anxiety. Each symptom is rated on a four-point scale from “0 = no” up to “3 = severe”, resulting in sum scores from 0 to 30 points (Table 1).

**Table 1:** Results of the MIDOS questionnaire

Symptom, *n* (%)	HFU (*n* = 137)	OC (*n* = 122)	*p*-value
*Pain*			0.012
None	71 (51.8)	83 (69.2)	
Mild	48 (35.0)	21 (17.5)	
Moderate	13 (9.5)	13 (10.8)	
Severe	5 (3.6)	3 (2.5)	
*Nausea*			0.441
None	109 (79.6)	105 (86.8)	
Mild	24 (17.5)	13 (10.7)	
Moderate	2 (1.5)	2 (1.7)	
Severe	2 (1.5)	1 (0.8)	
*Vomiting*			0.012*
None	123 (89.8)	118 (99.2)	
Mild	10 (7.3)	0 (0.0)	
Moderate	3 (2.2)	1 (0.8)	
Severe	1 (0.7)	0 (0.0)	
*Dyspnea*			0.001*
None	41 (29.9)	47 (38.8)	
Mild	22 (16.1)	35 (28.9)	
Moderate	41 (29.9)	31 (25.6)	
Severe	33 (24.1)	8 (6.6)	
*Obstipation*			0.001*
None	80 (58.8)	106 (86.9)	
Mild	39 (28.7)	10 (8.2)	
Moderate	11 (8.1)	4 (3.3)	
Severe	6 (4.4)	2 (1.6)	
*Weakness*			0.001*
None	22 (16.1)	43 (35.2)	
Mild	43 (31.4)	38 (31.1)	
Moderate	43 (31.4)	30 (24.6)	
Severe	29 (21.2)	11 (9.0)	
*Lack of appetite*		94 (77.0)	<0.001*
None	58 (42.3)	14 (11.5)	
Mild	42 (30.7)	11 (9.0)	
Moderate	24 (17.5)	3 (2.5)	
Severe	13 (9.5)		
*Fatigue*	29 (21.2)	32 (26.4)	0.043
None	40 (29.2)	46 (38.0)	
Mild	36 (26.3)	30 (24.8)	
Moderate	32 (23.4)	13 (10.7)	
Severe			
*Depression*			0.778
None	101 (73.7)	82 (68.3)	
Mild	21 (15.3)	24 (20.0)	
Moderate	12 (8.8)	11 (9.2)	
Severe	3 (2.2)	3 (2.5)	
*Anxiety*			0.689
None	86 (62.8)	82 (67.8)	
Mild	35 (25.5)	24 (19.8)	
Moderate	13 (9.5)	11 (9.1)	
Severe	3 (2.2)	4 (3.3)	

*HFU* heart failure unit, *OC* outpatient clinic

The MIDOS shows good internal consistency (Cronbach’s α = 0.723) [27]. In our study, Cronbach’s α was 0.803.

We used two psychometric scales to evaluate the psychological burden: The Distress Thermometer [28] (DT) and the 4-item Patient Health Questionnaire (PHQ-4) [29]. The DT is an 11-point visual analogue scale ranging from “0 = no distress” up to “10 = extreme distress”, self-assessing patients’ level of distress over the past week. A cut-off ≥ 5 indicates clinically significant levels requiring additional assessment and treatment [28, 30]. To identify potential sources of distress, a supplemental list of 37 problems had to be attributed with “yes” or “no”.

The PHQ-4 was used to screen for both anxiety and depressive disorders. Of its 4 items, 2 assess core depressive symptoms and two anxiety symptoms over the past 2 weeks. Items are scored on a four-point Likert scale from ‘0 = not at all’ to ‘3 = nearly every day’, thus PHQ-4 scores range from 0 to 12 [29]. Scores of 0 to 2 can be interpreted as ‘normal’, 3 to 5 as ‘mild’, 6 to 8 as ‘moderate’ (‘yellow flags’) and ≥ 9 as ‘severe’ symptoms of anxiety and depression (‘red flags’) [31]. With Cronbach’s α of 0.85, internal consistency of the PHQ-4 was high [29]. In our study, Cronbach’s α was 0.866.

### Screening for PC needs

Patients consecutively underwent a physician-directed screening for PC needs. The “Palliative Care Screening Tool for HF Patients” [32] (PCST), a modified version of the “Five-Item Palliative Care Screening Tool” [33], is a 9-item questionnaire for treating cardiologists/HF specialists querying objective indicators of additional multi-disciplinary palliative support in addition to standard HF management, which also includes the caregiver’s perspective (Table 2). PC needs are indicated by scores ≥ 5 out of a maximum of 12 points [34], while this cut-off has yet to be validated for the modified version [32]. In a previous study with HF patients, the internal consistency of the PCST was reported by Cronbach’s α as 0.580 [32]. In the present study, Cronbach’s α was 0.601.

**Table 2:** Results of the Palliative Care Screening Tool for HF Patients

Screening item*, n (%)*	Points	HFU (*n* = 137)	OC (*n* = 122)	*p*-value
Presence of chronic HF	1	137 (100)	122 (100)	
NYHA functional class	1–4			< 0.01*
I		21 (15.3)	16 (13.1)	
II		35 (25.5)	42 (34.4)	
III		50 (36.5)	55 (45.1)	
IV		31 (22.6)	9 (7.4)	
*Serious complications* of chronic heart failure (prognosis of < 12 months)	1	80 (58.4)	24 (19.7)	< 0.01*
*Serious comorbidity* associated with poor prognosis	1	109 (79.6)	52 (42.6)	< 0.01*
*Uncontrolled symptoms* despite cardiologic treatment	1	59 (43.1)	46 (37.7)	0.380
Moderate to severe *distress* in patient and/or family related to chronic heart failure diagnosis or therapy	1	91 (66.4)	13 (10.7)	< 0.01*
Patient and/or family *concern* about course of disease and decision making	1	91 (66.4)	23 (18.9)	< 0.01*
Patient and/or family *request palliative care consultation*	1	7 (5.1)	0 (0.0)	0.011*
Cardiologic team needs assistance with complex *decision making* or determining *goals of care*	1	7 (5.1)	2 (1.6)	0.128
Screening sum score*, M* ± *SD*	1–12	6.59 ± 2.2	4.81 ± 1.8	< 0.01*

### Statistical analyses

Descriptive statistics including frequencies, means, and standard deviations were determined, as well as medians with interquartile ranges (IQR) for not normally distributed variables. Group differences were analyzed using *T*-tests and χ^2^-tests or Fisher’s exact tests, according to the metric of the dependent variable. All significance tests were two-tailed using a significance level of α < 0.05. Analyses and graphs were performed or created using SPSS statistics software version 24.0 (IBM, USA).

## Results

### Demographic and clinical characteristics

**Fig. 1:**
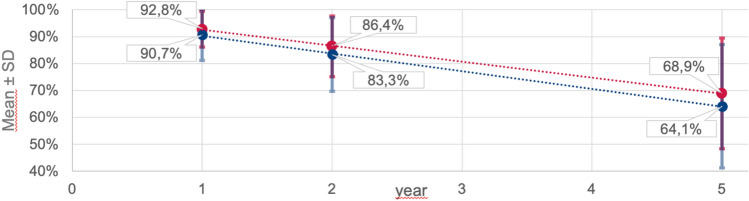
Projected survival in the Seattle Heart Failure Model. Abbreviations: *HFU* heart failure unit, *OC* outpatient clinic

A total of 259 patients were studied, 137 (53%) as inpatients at the HF unit (HFU) and 122 (47%) as outpatients at the specialised outpatient clinic (OC). There was a predominance of men in both study groups (HFU 69%, OC 75%, *p* = 0.225). Hospitalised patients were older (mean age 67.0 ± 13.9 vs. 57.9 ± 13.8 years, *p* < 0.01) and less frequently suffered from ischaemic cardiomyopathy, while most of the cardiac and non-cardiac comorbidities were evenly distributed between the two study groups (Table 3). More than half of the patients exhibited NYHA class III or IV symptoms (HFU 59% vs. OC 53%). The mean projected survival was better in the outpatient group each at one (92.8 ± 6.6% vs. 90.7 ± 9.5%), two (86.4 ± 11.3% vs. 83.3 ± 13.7%), and five years (68.9 ± 20.6% vs. 64.1 ± 23.0%) (Fig. 1).

**Table 3:** Patient characteristics

	HFU (*n* = 137)	OC (*n* = 122)	*p*-value
*Clinical variables*			
Age, *M* ± *SD (range)*	67.0 ± 13.9 (18–90)	57.9 ± 13.8 (19–83)	< 0.01**
Male gender, *n (%)*	94 (68.6)	92 (75.4)	0.225
*Aetiology of heart disease, n (%)*			
Dilated cardiomyopathy	52 (38.0)	43 (35.2)	< 0.01**
Ischaemic cardiomyopathy	11 (8.0)	61 (50.0)	
Valvular cardiomyopathy	40 (29.2)	11 (9.0)	
Others/unknown	34 (24.8)	7 (5.7)	
*History of comorbidities, n (%)*			
Arterial hypertension	69 (50.4)	61 (50.0)	0.953
Diabetes	46 (33.6)	28 (23.0)	0.059
Obesity	20 (14.6)	42 (34.4)	< 0.01**
Chronic renal failure	52 (38.0)	30 (24.6)	0.021*
Peripheral artery disease	46 (33.6)	14 (11.5)	< 0.01**
Malignancy	26 (19.0)	16 (13.1)	0.201
Pulmonary disease	33 (24.1)	37 (30.3)	0.259
Neurologic comorbidity	22 (16.1)	24 (19.7)	0.448
Hypothyroidism	13 (9.5)	18 (14.8)	0.193
Hyperthyroidism	8 (5.8)	9 (7.4)	0.618
Psychiatric/psychological comorbidity	13 (9.5)	9 (7.4)	0.543
Nicotine abuse	32 (23.4)	43 (35.2)	0.035*
Alcohol abuse	7 (5.1)	6 (4.9)	0.944
*Cardiac history and presentation, n (%)*			
Pacemaker/intracardiac device	23 (16.8)	78 (63.9)	< 0.01**
Prior cardiac surgery	35 (25.5)	40 (32.8)	0.200
History of CPR	6 (4.4)	6 (4.9)	0.837
History of atrial fibrillation	59 (43.1)	46 (37.7)	0.380
Peripheral edema upon presentation	50 (36.5)	18 (14.8)	< 0.01**
NYHA I	21 (15.3)	16 (13.1)	< 0.01**
NYHA II	35 (25.5)	42 (34.4)	
NYHA III	50 (36.5)	55 (45.1)	
NYHA IV	31 (22.6)	9 (7.4)	
*Eurotransplant waiting list status, n (%)*			
Listed “transplantable” (T-status)	3 (2.2)	9 (7.4)	0.047*
Listed “high-urgency” (HU-status)	4 (2.9)	0 (0.0)	0.057

*HFU* heart failure unit, *OC* outpatient clinic, *M* mean, *SD* standard deviation, *NYHA* New York heart association, *CPR* Cardiopulmonary resuscitation

### Patient-reported outcome measures

**Fig. 2:**
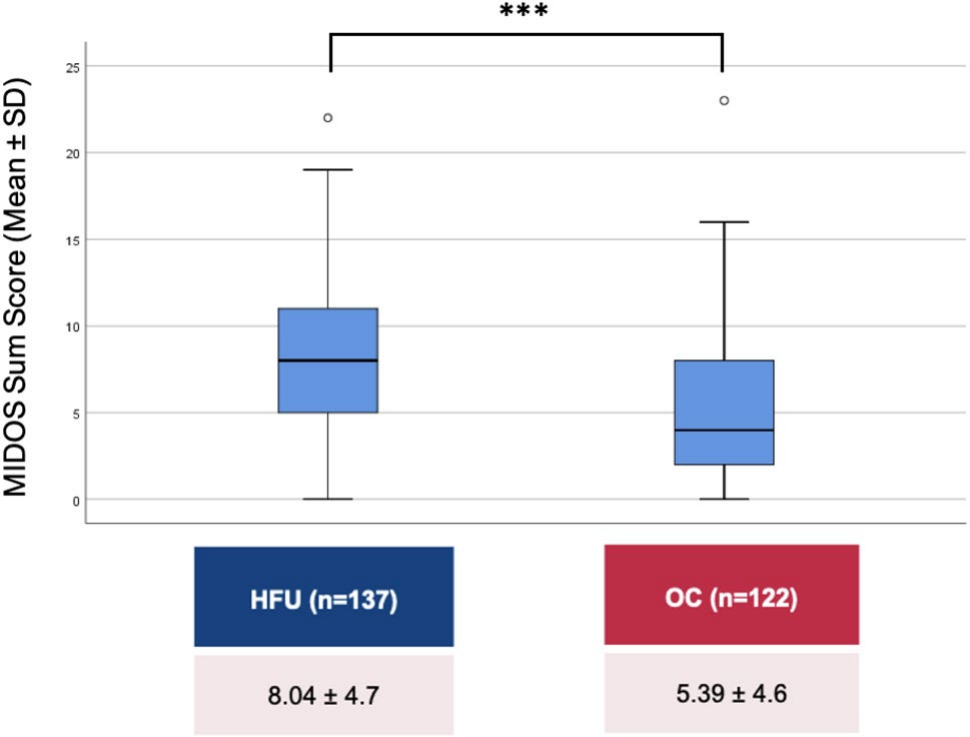
Results of the MIDOS questionnaire. Abbreviations: *HFU* heart failure unit, *OC* outpatient clinic, *MIDOS* minimal documentation system for palliative care, *M* mean, *SD* standard deviation

The MIDOS questionnaire revealed a more severe symptom burden among hospitalised patients, among whom mean MIDOS scores were 8.0 ± 4.7, compared to 5.4 ± 4.6 in HF outpatients (max. 30 points, *p* < 0.001, Table and Fig. 2). In both groups, dyspnea, fatigue and weakness were the 3 most frequent of at least “moderate” intensity, while dyspnea and fatigue were experienced more often by hospitalised patients.

**Fig. 3:**
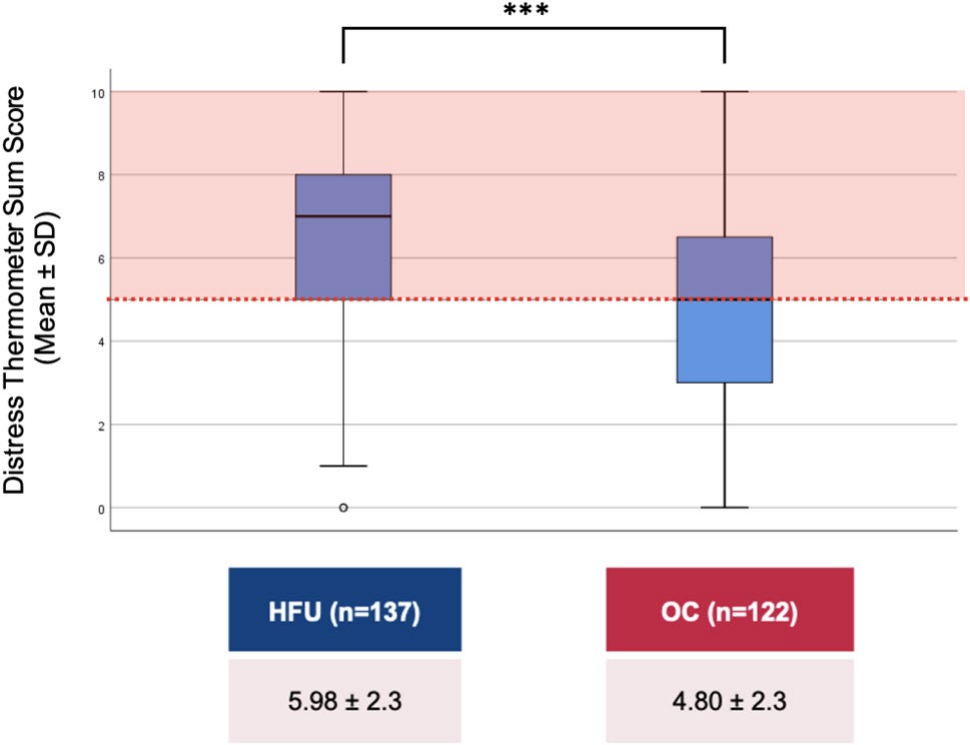
Results of the distress thermometer. Abbreviations: *HFU* heart failure unit, *OC* Outpatient clinic, *M* mean, *SD* standard deviation

Mean Distress Thermometer (DT) scores were 6.0 ± 2.3 in inpatients vs. 4.8 ± 2.3 in outpatients (max. 10 points, *p* < 0.001, Fig. 3). 75.7% of HFU patients compared to 56.7% of OC patients (*p* = 0.001), experienced clinically relevant levels of distress (as indicated by DT scores ≥ 5). The three most frequently reported problems in both groups were fatigue, breathing, and sleeping problems. Emotional problems, family problems, and problems of practical nature, as well as spiritual concerns, were considered less relevant compared to physical complaints.

The PHQ-4 screening did not show differences between in- and outpatients (mean PHQ-4 score 2.3 ± 2.5 in inpatients vs. 2.2 ± 2.5 in outpatients, *p* = 0.78). 40.9% of in- and 35.5% of outpatients reported *mild* symptoms of depression or anxiety (scores ≥ 3), and *moderate* or *severe* symptoms were detected in 13 HFU (9.5%) and 12 OC patients (9.8%, *p* = 0.275).

### Screening for PC needs

**Fig. 4:**
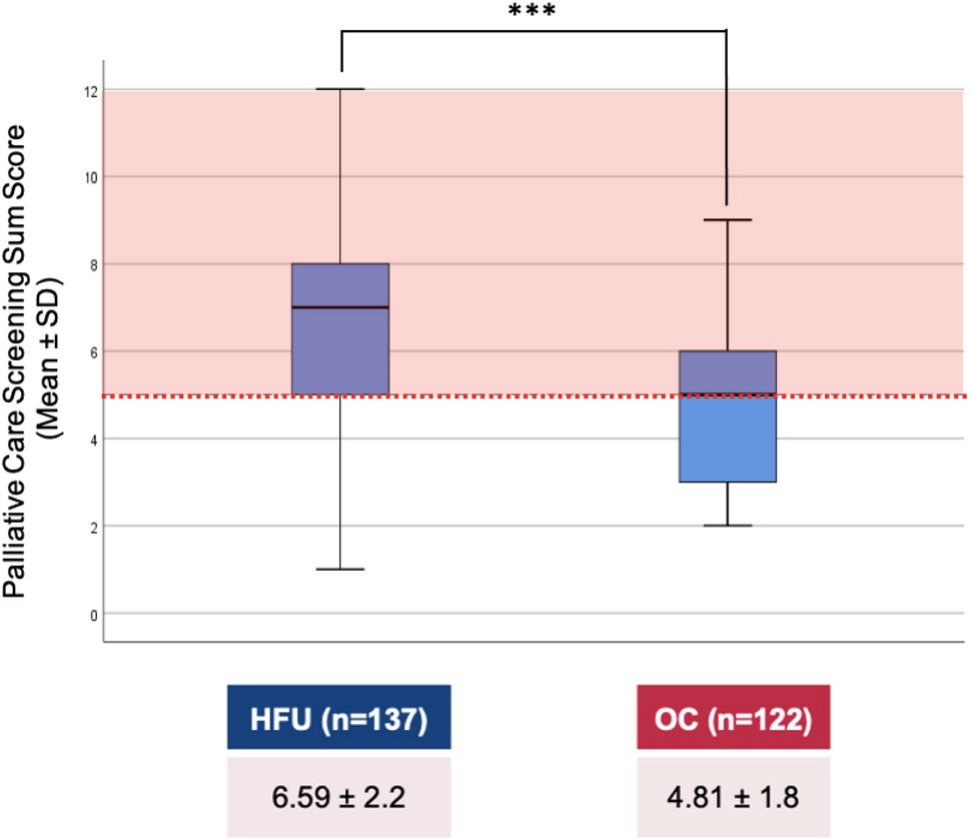
Results of the palliative care screening tool for HF patients. Abbreviations: *HFU* heart failure unit, *OC* outpatient clinic, *M* mean, *SD* standard deviation

The physician-directed screening for PC needs showed a higher mean score in the group of hospitalised patients (PCST mean score 6.6 ± 2.1 vs. 4.8 ± 1.8, *p* < 0.01, Table 2; Fig. 4). Assuming scores ≥ 5 to indicate a need for palliative support, four out of five inpatients (82.5%) and more than half of outpatients (52.5%) studied would require additional attention in this regard. Irrespective of this, the screening revealed that only seven hospitalised patients (5.1%) – and not a single patient in the outpatient group – had actively requested a PC consultation, and the cardiologic team rarely (in 3.4% of all patients) saw itself in need of specialist assistance, e.g., with complex decision making or determining the goals of care.

## Discussion

In a cohort of HF patients at a specialized service, a high symptom burden and level of psychosocial distress were detected using a novel cross-sectional approach. Many HF patients fulfil the criteria for “specialised PC”, which—given the relatively good life expectancy of these patients—may indicate a need for tailored comprehensive or holistic care, rather than PC in its traditional sense.

Design and broad inclusion criteria of this pilot study led to the creation of a heterogeneous sample of HF patients with a comparably young age and good survival prognosis. Compared to the PAL-HF population [24], patients in the study were younger, had fewer comorbidities and a lower NYHA functional class. This may represent the actual clinical situation in a specialized HF service better than a highly selected sample of the “sickest of the sick” patients with terminal HF or in “end-of-life situations”, who have traditionally been the target population for assessing PC needs. One may argue that “PC needs” in this “well-off” HF population rather represent “general needs for holistic care”, even though the PCST was adapted from an instrument used to screen for PC needs in advanced cancer patients [33] and the other patient-reported outcome measures (PROMs) of this study are routinely used in PC assessments in Germany as well [35, 36]. It may be challenging to distinguish differential indications for PC from other person-centered modalities of care (e.g., psycho-cardiological treatment, psychosocial treatment, or collaborative care), especially given the relatively long projected survival in our cohort and the possible effects on cardiac outcomes of such interventions [37].

Comparing results of the current study with previously published studies from our tertiary care centre may help put the findings into perspective. PROMs employed in this study were previously applied in advanced HF patients on LVAD support and heart transplant recipients [38] and, naturally, terminal cancer inpatients [39]. While heart transplant recipients have the lowest MIDOS score [38], which can be expected and attributed to the nature of the received treatment, the MIDOS score of terminal cancer patients exceeds those of HF patients [39], even of those hospitalized. However, before prematurely concluding that the symptom burden in terminal cancer may ultimately be higher than in advanced HF, it should be taken into consideration that the MIDOS questionnaire may not ideally portray HF symptomatology. According to previous studies on HF-specific PROMs, HF patients mostly exhibit three “hallmark symptoms”—dyspnea, fatigue, and weakness [40, 41]—which was also observed in the presented study. In contrast, cancer patients, as the “original” target population of the MIDOS questionnaire, usually suffer from a larger variety of symptoms, thus resulting in higher MIDOS scores. Especially gastrointestinal symptoms, which play a subordinate role in HF, seem to be over-represented in the MIDOS, which may consequently be a less favourable instrument for assessing symptom burden in HF than widely established and evidence-based disease-specific PROMs as the Kansas City Cardiomyopathy Questionnaire (KCCQ) or the Minnesota Living with Heart Failure Questionnaire (MLHFQ).

The psychosocial burden and psychological co-morbidities in advanced HF may frequently be underestimated or even neglected [42, 43]. Many PRO instruments lack psychometric properties, and the KCCQ and MLHFQ have limited focus on emotional and relationship problems [12]. Comparing levels of distress, hospitalized HF patients in the study had DT scores almost equivalent to those of patients with terminal cancer [39]. Besides, independent of this treatment setting, a high prevalence of clinically relevant distress in HF was detected, and correspondingly a high number of patients exhibited *mild* symptoms of depression and anxiety. Depression and anxiety disorders are the most common psychiatric comorbidities in HF with a prevalence of up to 30% [44], and have been shown to inversely affect prognosis [44, 45]. In the study, the prevalence of “yellow” and “red flags” in the PHQ-4 screening (*moderate* or *severe* symptoms) was much lower, so depression and anxiety may predominantly be limited to a “sub-syndromal” level.

The PCST showed that many HF patients fulfil criteria for “specialised PC”. Across different HF cohorts, LVAD patients, together with hospitalized HF patients, may have the greatest need for additional supportive treatment [38]. While benefits of PC interventions in HF have recently been demonstrated, evidence that patients judged as “being in need of PC” by the PCST profit from such care is so far lacking. First, the patient’s and caregiver’s perspective on such treatment should be obtained, and a specialist consultation to ascertain the indication for PC be arranged.

Apart from the MIDOS, the tools used in this study’s cross-sectional approach could be a valuable addition to the assessment of PROMs in HF. They moreover proved easy and self-explanatory in use, allowing for assessments to be smoothly integrated into clinical routine in the setting of specialist HF care, which is in line with previous studies [32, 38].

**Fig. 5:**
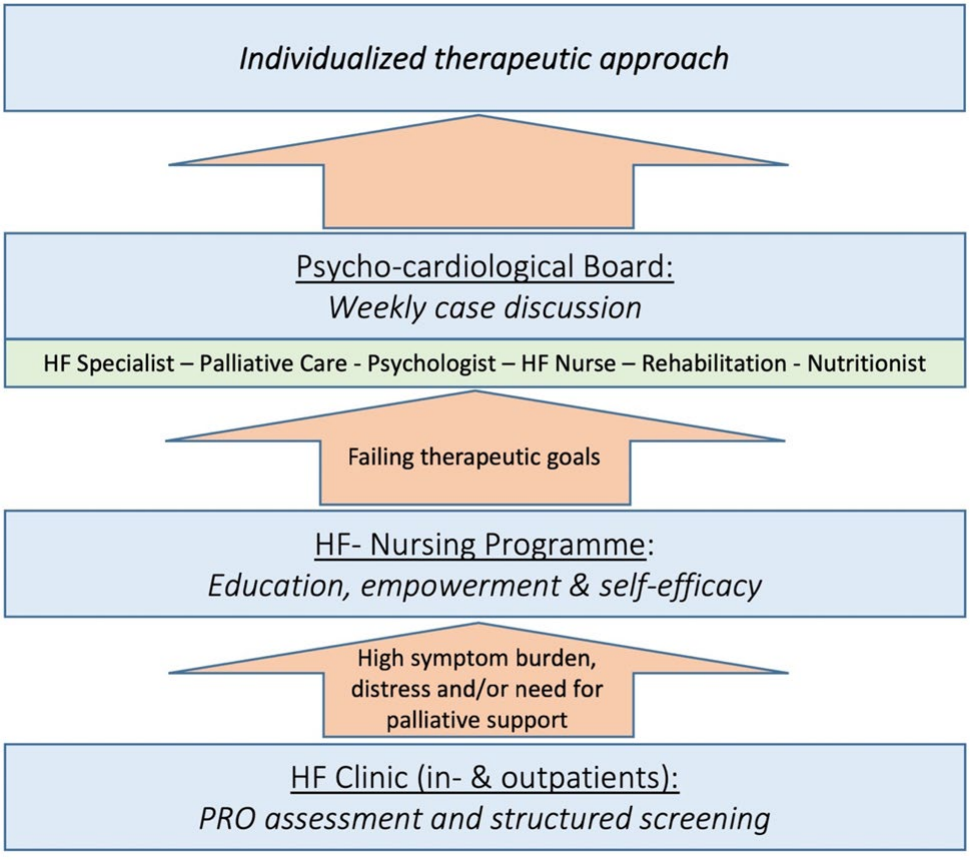
Proposal of a stepwise approach to holistic HF care

As a consequence of this study’s observations, a multi-disciplinary stepwise approach to improve holistic HF care was implemented at our institution, which is based on the structured application of the PCST, DT, and a set of validated PROMs recommended by the International Consortium for Health Outcomes Measurement (ICHOM) in HF patients: The KCCQ-12, PROMIS (Patient-Reported Outcome Measurement Information System), and PHQ-2 [46]. Patients with a high symptom burden, high level of distress or suspected needs for PC are taken up into a nurse-led HF education and counseling programme, focusing on strengthening patient resources, knowledge building, and self-efficacy. If ongoing distress is observed and treatment goals are not met, patients’ cases are discussed in an interdisciplinary psycho-cardiological board deciding on an individualized therapeutic approach. Also, a direct, low-barrier PC specialist consultation for patients with a positive screening for PC needs can be initiated (Fig. 5).

### Study limitations

This study has several limitations. While this analysis used patients prospectively enrolled at a large tertiary HF centre, the single-centre nature of the data and the small sample size are limitations that hamper generalizability of the presented findings. There are moreover methodological limitations owing to the explorative, pilot character of this observational study and the used assessment instruments.

The study design can only insufficiently capture the characteristic symptom trajectory of HF. Especially patients’ long-term needs may be inadequately examined, as symptom burden, psychosocial distress, and PC needs usually improve after discharge. Instead, longitudinal data from a “staged” design with assessments during both hospitalization *and* after discharge could be a preferable approach.

Concerning the PROMs used in the study, the MIDOS questionnaire may not be suitable to properly portray the symptom burden in HF, and thus be inferior to other well-established HF-specific PROMs in this respect. Further limitations concern the internal consistency of the PCST. Cronbach’s α was 0.601, a value lower than considered to be adequate (α ≥ 0.70). This could be due to the low number of items, poor item inter-relatedness or heterogenous constructs, and warrants further research to assess the dimensionality and underlying structure of the PCST. As the study’s PROMs are not well established in HF, assessment of a disease-specific QoL questionnaire as a comparator would have been of added value.

Finally, conclusions established from pooled data from in- and outpatients should be regarded as hypothesis-generating and call for independent validation to mitigate these limitations. A randomized-controlled follow-up trial investigating the benefit of structural screening for PC needs using well-chosen PROMs and tailored multi-professional PC interventions could validate this study’s finding and further underline the paramount importance of holistic care in HF.

## Conclusion

Patients with advanced HF, even though with relatively good prognosis, have multiple needs that are often not detected and addressed before “truly palliative” treatment situations are reached. Next to improving prognosis, patients’ well-being should be accepted as a primary goal of care, as many patients prefer a better QoL to prolongation of life [10, 11].

The tools in this study seem useful to explore unmet needs, and the screening results may help to improve integrated HF care through a nurse-led HF education and counseling programme. Longitudinal studies in larger well-defined cohorts are required to investigate whether these effects can really be achieved using such interventions.
